# The impact of digital channels on public health services to enhance city resilience during the public health emergency response in Thailand (2020–2023)

**DOI:** 10.1186/s12913-025-13480-4

**Published:** 2025-09-30

**Authors:** Watcharaporn Chutarong, Roongaroon Thammalikhit, Anurak Sawangwong, Yuqian Guo, Muhammad Fawad, Supaporn Lonapalawong, Weiwen Zhang

**Affiliations:** 1 Department of Urban Development and Management, School of Public Affairs, Zhejiang University, Hangzhou, Zhejiang China; 2 School of Public Affairs, China Institute of Urbanization, Zhejiang University, Hangzhou, Zhejiang China; 3 The Division of Preventive Medicine, Directorate of Medical Services, Royal Thai Air Force, Bangkok, Thailand; 4 Industrial Engineering, Faculty of Engineering, Thai-Nichi Institute of Technology, Bangkok, Thailand; 5 Department of Anesthesiology and Intensive Care Medicine, The First Affiliated Hospital, School of Medicine, Zhejiang University, Hangzhou, Zhejiang China; 6 Unit of Health Sciences, Faculty of Social Sciences, Tampere University, Tampere, Finland; 7 School of Mathematics and Computer Science, Yunnan Minzu University, Kunming, Yunnan China

**Keywords:** Public health service, Digital channel, City resilience, Public health emergency, Infectious disease control, Public health policy

## Abstract

**Background:**

This study examines the impact of digital channels on public health services and their role in enhancing city resilience.

**Methods:**

Conducted in Thailand, this study employed an online questionnaire distributed in both Thai and English from December 2023 to February 2024. A total of 824 valid responses from Thai nationals and foreigners were analyzed. Exploratory factor analysis, confirmatory factor analysis, and structural equation modeling were performed using IBM SPSS 23 and AMOS 23.

**Results:**

Digital channels have a strong positive direct effect on public health services (*β* = 0.70, *p* < 0.001), public health services have a strong positive direct effect on resilient cities (*β* = 0.69, *p* < 0.001), and digital channels have a low positive direct and a moderate positive indirect effect on resilient cities (*β* = 0.19, *p* < 0.001, and *β* = 0.48, *p* < 0.001, respectively). The model demonstrated excellent fit with the empirical data: *X*^*2*^/*df* = 2.189, CFI = 0.954, GFI = 0.900, NFI = 0.919, TLI = 0.946, RMSEA = 0.038, and RMR = 0.047.

**Conclusions:**

Digital channels are critical tools for collecting, analyzing, and disseminating public health service data. They improve public health services across five dimensions—product, facility, service, process, and price—while also strengthening city resilience in disaster management, economic stability, quality of life, and institutional capacity.

**Supplementary Information:**

The online version contains supplementary material available at 10.1186/s12913-025-13480-4.

## Background

Throughout history, humanity has faced numerous public health emergencies (PHEs). Several outbreaks—including H1N1 (2009–2010), poliovirus (2014–2020), Ebola in West Africa (2014–2016), Zika virus (2016), Ebola in the Democratic Republic of the Congo (2019–2020), and coronavirus disease 2019 (2019–2023)—have been declared public health emergencies of international concern (PHEICs) by the World Health Organization (WHO) [1]. Infectious diseases, as a persistent threat to global health, have caused significant mortality across decades. The most recent pandemic, coronavirus disease 2019, resulted from severe acute respiratory syndrome coronavirus 2 (SARS-CoV-2) [2]. Beyond endangering public health, the virus’s rapid transmission disrupted socioeconomic systems, leading to travel bans, job losses, and business closures [3]. These impacts underscore the critical need for resilient public health services to mitigate such crises [4].

To mitigate disease transmission, individuals need to recognize the seriousness of the illness and understand appropriate preventive measures. Rosenstock (1974) outlined the core principles of the Health Belief Model (HBM), proposing that individuals adopt disease-preventing behaviors only when they perceive themselves as susceptible to the disease, acknowledge its potential severity and impact on their lives, and believe their actions can effectively reduce risk or mitigate consequences—provided no psychological barriers (e.g., cost, inconvenience, or stigma) outweigh these perceptions [5]. Becker (1974) later expanded this model by incorporating additional determinants of preventive behavior, including perceived benefits, cues to action, and health motivation [6]. These theoretical foundations underscore the importance of disseminating accessible public health information during outbreaks. By raising awareness of available services and disease-specific guidance, health authorities can foster the adoption of protective behaviors in at-risk populations [7].

Public health services encompass medical and health-related interventions implemented during disease outbreaks, PHEs, healthcare management initiatives, or health promotion campaigns [8]. Previous study has established that effective delivery of public health services is critical for combating infectious diseases [9]. As Stoto et al. (2017) emphasize, these services are vital not only for addressing immediate casualties but also for facilitating recovery and restoring normalcy [10]. Key components include providing vaccinations, distributing medical supplies, and delivering physical and mental healthcare to victims of PHEs [10]. Furthermore, such services incorporate preventive measures like immunization programs [9, 10]. Ultimately, robust public health service delivery forms the foundation of health system development [11].

PHEs demand robust and well-coordinated response systems to mitigate their impact on populations. Extensive research has identified five critical dimensions that determine the effectiveness of public health service delivery during such crises. These interlinked components form the foundation for successful emergency response and recovery efforts. First, the product dimension encompasses the tangible goods necessary to address urgent health needs during emergencies [12, 13]. As documented in recent studies, the availability and quality of essential medical products directly influence the success of public health interventions [12, 13]. These products range from basic protective equipment like masks and hand sanitizers to more complex medical resources such as vaccines, therapeutic medications, and diagnostic tools including antigen test kits (ATKs) and reverse transcription-polymerase chain reaction (RT-PCR) equipment [12–14]. The timely provision of appropriate products can significantly enhance the overall effectiveness of public health responses to crises. The second crucial dimension, price, addresses the economic aspects of service delivery [15–17]. Some studies emphasizes that the affordability and fairness of costs for both goods and services play a pivotal role in emergency response [15–17]. This dimension includes not only the direct healthcare expenses but also accounts for diversity-related costs and the availability of various payment options [15–17]. Equitable healthcare provision during crises requires the removal of financial obstacles, ensuring marginalized populations maintain consistent access to essential services [18]. Equally important is the service dimension, which encompasses both tangible and intangible support provided to affected population [19]. Beyond the physical medical interventions, this dimension includes critical services such as counseling and comprehensive outreach programs focused on disease prevention and control. These services often prove vital in maintaining public trust and compliance with health measures during prolonged emergencies [19]. The process dimension represents the operational framework that guides how services are accessed and delivered [15, 20, 21]. This includes clear communication of service information, efficient registration procedures, and standardized data collection protocols. Well-designed processes ensure that limited resources are optimally utilized and that beneficiaries can navigate the system without unnecessary barriers [15, 20, 21]. Finally, the facility dimension concerns the physical infrastructure supporting service delivery [22]. Hospitals, quarantine centers, and isolation facilities require strategic geographic distribution and sufficient resource allocation to effectively serve population demands. Geographic accessibility of these facilities is particularly crucial during emergencies when mobility may be restricted and timely access to care can significantly affect outcomes [22]. These five dimensions - product, price, service, process, and facility - collectively form an integrated framework for effective public health service delivery during emergencies.

The recent PHE response (2022–2023) highlighted significant challenges in delivering public health services during PHEs, particularly when maintaining physical distancing between providers and affected individuals was crucial [23–25]. These challenges underscore the critical role of digital channels in public health service delivery during such crises. Digital channels—defined as the online platforms, tools, and techniques used for communication, engagement, and service delivery—facilitate the exchange of diverse content, including documents, audio, video, images, and informational resources [26]. Examples include websites, mobile applications, social media platforms, and television channels [24, 27–29]. Empirical studies have demonstrated their utility in disseminating information and delivering public health services [30–32]. During the SARS-CoV-2 pandemic (2020–2023), governments leveraged these channels extensively to raise awareness about transmission risks through mass communication campaigns and collect and analyze health and personal data from the public to inform response efforts [28]. This adoption of digital solutions not only addressed physical distancing constraints but also enhanced the efficiency and reach of public health interventions [32].

Similar to resilient cities, public health services during PHEs aim to restore normalcy as efficiently as possible [10, 33]. Resilient cities—defined as city systems capable of planning for, absorbing, adapting to, and recovering from adverse events like natural disasters, economic crises, and PHEs [3, 34]—maintain operational continuity across four key domains: institutional governance, disaster response systems, economic activity, and daily community life [35]. Digital channels play a pivotal role in this process by enabling bidirectional data exchange between public health providers and recipients. These platforms facilitate effective risk communication, health information aggregation, and real-time data generation, ultimately enhancing city resilience until full societal recovery is achieved [36, 37].

Thailand’s public health service delivery during the PHE from 2020 to 2023 encountered significant practical challenges, including delayed public access to service information, inadequate reach to target populations, and widespread misinformation (“fake news”) on social media platforms [9, 38, 39]. This underscores the government’s imperative to enhance public communication strategies, ensuring both message consistency and comprehensive population coverage. At the theoretical level, the literature reveals three persistent theoretical problems regarding digital channels’ role in PHE responses. First, significant mechanistic uncertainty exists, as current research fails to adequately explain how digital channels actively support public health services and contribute to city resilience [30, 40]. While studies have identified relationships between these elements, they offer limited insight into public health services’ distinct functional contributions or the tripartite dynamics between digital infrastructure, health services, and resilience outcomes [36, 40, 41]. Second, the field lacks consensus on both the determinants of public health service effectiveness and their validated measurement indicators [10, 42]. Third, insufficient studies examined which specific city functions (e.g., economic, institutional) are susceptible to public health interventions’ effects [33, 43].

Addressing these below gaps is essential for developing evidence-based PHE response frameworks. On a practical level, the Thai government still lacks assessments of how public health policies impact city resilience, and the role of digital channels in public health service delivery during PHEs remains underexplored. Furthermore, empirical studies often lack sufficient primary data to evaluate public health service performance [9, 44].Theoretically, further research is needed to clarify the tripartite relationship between digital channels, public health services, and resilience cities [36, 41]. Additionally, existing public health service guidelines are sparse and inconsistently applied during PHEs, necessitating clearer systematic identification of core dimensions and measurable indicators [4, 45]. Finally, additional studies are necessary to clarify the city functions influenced by public health policy implementation [33, 43].

This study examines the impact of digital channels on public health services and their role in enhancing city resilience, with particular relevance to Thailand’s SARS-CoV-2 response (2020–2023). The study makes three significant contributions for public health policy planners: first, a comprehensive framework of public health service indicators and dimensions to enhance pandemic response efficiency [9, 18, 44]; second, actionable recommendations to improve municipal operations through measurable resilience dimensions and their indicators [3, 33]; and third strategic guidance for leveraging digital channels to optimize public communication and crisis data management systems [46]. Through a causal relationship model, this study examines the relationships between three primary latent variables: digital channels, public health services, resilient cities. The specific sub-objectives are: (1) to examine the impact of digital channels on public health services; (2) to examine the impact of public health services on resilient cities; and (3) to examine the impact of digital channels on resilient cities.

## Methods

### Instrument

This study utilized a structured self-administered questionnaire to evaluate the relationship between digital channels, public health services, and resilient cities during Thailand’s PHE response (2020–2023). The instrument comprised four distinct sections: (1) basic information, (2) digital channels (4 items), (3) public health services (32 items), and (4) resilient cities (16 items focusing specifically on government-declared easing of control measures). Sections 2 through 4 employed a standardized 5-point Likert scale, where participants indicated their level of agreement (1 = strongly disagree to 5 = strongly agree) with carefully developed statements assessing each construct [47]. Higher composite scores on this 52-item instrument reflected stronger perceived effectiveness of digital channels in service delivery, greater perceived effectiveness of public health services overall, and more positive assessments of city resilience outcomes during the pandemic period. The questionnaire was specifically designed to capture both the role of digital channels in public health service delivery and their cumulative impact on building city resilience throughout Thailand’s SARS-CoV-2 response.

This study operationalizes three core concepts: (1) Digital channels denote the online platforms, tools, and communication methods that enable the dissemination of multimedia content (documents, audio, video) and service delivery [26]. Digital channels encompass diverse platforms such as websites, mobile applications, social media platforms, and television channels [27–29]. (2) Public health services refer to medical interventions and health programs implemented during disease outbreaks, PHEs, or health promotion initiatives [8]. (3) Resilient cities represent city systems capable of anticipating, absorbing, adapting to, and recovering from crises—including PHEs, economic shocks, and natural disasters—while maintaining essential functions. These interconnected concepts form the theoretical foundation for examining Thailand’s pandemic response [3, 34].

### Questionnaire validity and reliability

**Fig. 1:**
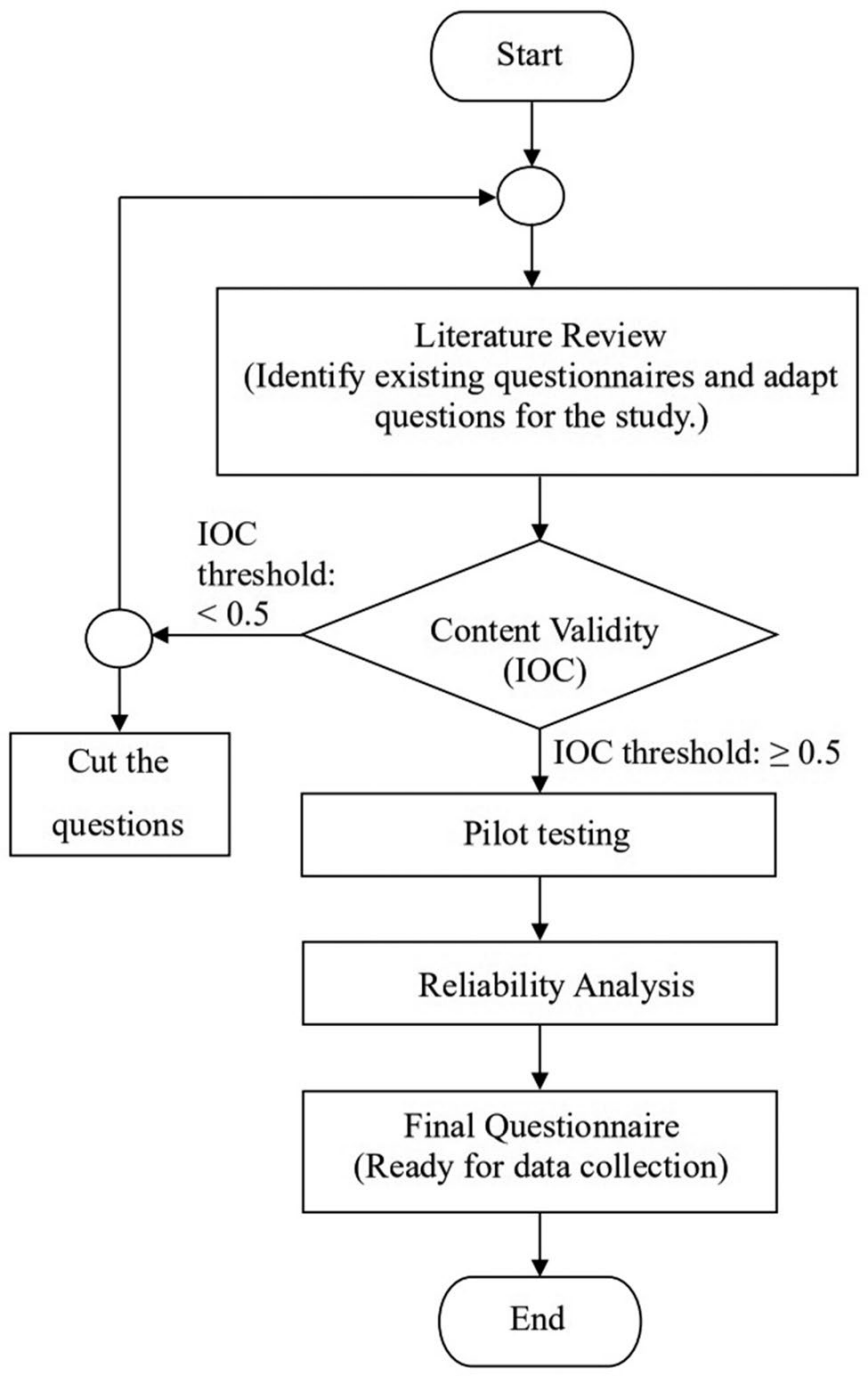
Questionnaire development flowchart

Prior to data collection, the questionnaire underwent rigorous validity and reliability testing. The development process (Fig. 1) began with a comprehensive literature review, with questions adapted from existing instruments (Table 1). Content validity was assessed using the Index of Item-Objective Congruence (IOC), evaluated by a multidisciplinary panel of three experts (public health, engineering, and political science) who rated question relevance on a + 1 (suitable), 0 (uncertain), or -1 (unsuitable) scale [48, 49]. All questions achieved an IOC of 1.00, exceeding the 0.5 threshold for satisfactory content validity [50]. Following content validity assessment, the questionnaire was distributed to a pilot sample through an online platform (Facebook and Line). The pilot study adhered to established methodological guidelines, incorporating two key considerations: (1) Connelly’s (2008) recommendation of 5–10% of the anticipated total sample size [51], and (2) Johanson and Brooks’ (2010) suggestion of a minimum of thirty representative participants from the target population for preliminary surveys [52]. This study’s pilot test involved 54 participants, representing 6.55% of the projected total sample size, thereby satisfying both methodological requirements while maintaining practical feasibility. For reliability testing, a pilot study was conducted with 54 participants, adhering to recommended guidelines for pilot sample sizes [51, 52]. Cronbach’s α coefficients confirmed high internal consistency across all primary latent variables: digital channels (α = 0.852), public health services (α = 0.900), and resilient cities (α = 0.799), with all values surpassing the 0.7 reliability benchmark [53].

**Table 1:** Construct and measurement items

Items	Indicators	References
DC1	Websites [27]	Zhong et al., 2021
DC2	Social Media Platforms [28]	Li et al., 2022
DC3	Mobile Applications [24]	Singh et al., 2020
DC4	Television Channels [29]	Nagata et al., 2022
PHS1	Information about medical supplies (masks and alcohol for hand sanitizers) [12]	Fairgrieve et al., 2020,
PHS2	Information about vaccines [12]	Fairgrieve et al.,2020,
PHS3	Information about medicines [12]	Fairgrieve et al., 2020,
PHS4	ATK [12]	Fairgrieve et al., 2020
PHS5	Information about medical equipment for RT-PCR testing [12]	Fairgrieve et al., 2020
PHS6	Information about service prices [17]	Chana et al., 2021
PHS7	Information about medical product prices [16]	Jain and Jain, 2022
PHS8	Information about available options to receive treatment or other services [15]	Ravangard et al., 2020
PHS9	Information about available choices of treatment prices or other services prices such as quarantine [16]	Jain and Jain, 2022
PHS10	Information about payment methods [27]	Zhong et al., 2021
PHS11	The infection test sites are available across the country [54]	Mehmood et al., 2024
PHS12	Vaccination sites are available across the country [4]	McCarthy et al., 2023
PHS13	Pharmacies and medicine stores are available across the country [55]	Jones-Jack et al., 2024
PHS14	Treatment facilities/hospitals are available across the country [45]	Yan et al., 2022
PHS15	Quarantine facilities are available across the country [12]	Wang et al., 2021
PHS16	Publicizing knowledge about vaccination, treatment, and self-protection [27]	Zhong et al., 2021
PHS17	Using social media platforms to provide information about virus infection [27]	Zhong et al., 2021
PHS18	Using mobile applications to increase communication channels [27]	Zhong et al., 2021
PHS19	Promoting or campaigning for vaccination/infection testing [46]	Habib et al., 2023
PHS20	Updating of public health service delivery quality (performance) [15]	Ravangard et al., 2020
PHS21	Accessing information, advice, or suggestions from volunteers [27]	Zhong et al., 2021
PHS22	Accessing information, advice or suggestions from government doctors or nurses [56]	Car et al., 2020
PHS23	Accessing information, advice or suggestions from non-government/government pharmacists [57]	Zheng et al., 2021
PHS24	Accessing information, advice or suggestions from government officials from other agencies [15]	Ravangard et al., 2020
PHS25	Registration (book an appointment) for vaccines/treatments/services [20]	Hindi et al., 2019
PHS26	Steps for receiving vaccines/treatments/services [21]	Mutia and Pujiyanto, 2022
PHS27	Details about recommendations of before and after of vaccination/treatments/services [15]	Ravangard et al., 2020
PHS28	Accessing personal health information [20]	Hindi et al., 2019
PHS29	Signs/logos of infection test facilities/vaccination facilities/treatment facilities/quarantine facilities [17]	Chana et al., 2021
PHS30	Internal and external environment of the healthcare facilities [58]	Capolongo et al., 2020
PHS31	Medical equipment in the healthcare facilities [59]	Nelson et al., 2021
PHS32	Details, pictorial, or video information on mobile apps/websites provided by the government [27]	Zhong et al., 2021
RC1	Ability to work/study online [60]	Xu et al., 2023
RC2	Ability to travel or utilize public transportation [60]	Xu et al., 2023
RC3	Ability to access the internet [34]	Kuo et al., 2022
RC4	Ability to employ water, electricity, gas, and fuel [34]	Kuo et al., 2022
RC5	The organizations can continue their work [61]	Suleimany et al., 2022
RC6	The organizations can cooperate with other organizations [61]	Suleimany et al., 2022
RC7	The government can continue responding to the PHE [61]	Suleimany et al., 2022
RC8	The coordination of the central government and other government agencies in responding to the PHE [61]	Suleimany et al., 2022
RC9	Ability to buy/sell products or services online [62]	Che et al., 2023
RC10	Ability to do financial transaction online [62]	Che et al., 2023
RC11	Ability to receive salary or income [61]	Suleimany et al., 2022
RC12	Having food, drinking water, clothing, medicine, and housing [61]	Suleimany et al., 2022
RC13	Following the disease prevention and control measures recommended by the government [63]	Champlin et al., 2023
RC14	Ability to communicate and do social activities through online (such as seminar, meeting, or other online activities) [63]	Champlin et al., 2023
RC15	Ability of people in sharing knowledge or information about self-protection to others [61]	Suleimany et al., 2022
RC16	Ability to access learning resources online [61]	Suleimany et al., 2022

Note. DC, digital channels; PHS, public health services; RC, resilient cities

### Study area

This study focuses on Thailand, a Southeast Asian nation situated at approximately 15°N latitude and 100°E longitude. With a land area of 513,120 km² and a population approaching 72 million [42], the country administratively consists of 77 provinces, including the capital region of Bangkok. Thailand experiences tropical climatic conditions, with annual temperatures ranging from 18 °C to 38 °C. The study particularly considers three strategically important city centers: Bangkok (the national economic and political hub), Chiang Mai (the northern cultural and historical center), and Phuket (a key maritime tourism and transportation gateway in the south).

### Study participants and sampling

This study employed an online survey methodology using Google Forms, disseminated through social media platforms (Facebook and Line) between December 2023 and February 2024. The questionnaire was developed in parallel Thai and English versions, with the English version translated from the original Thai instrument. Participants included both Thai nationals and foreign residents (visitors and immigrants) who had resided in Thailand for at least six months during the study period (3 January 2020–5 May 2023), from Thailand’s early case detection phase to the WHO’s declaration ending the PHEIC [43, 64]. Using convenience and snowball sampling techniques, recruitment occurred through social media networks, excluding individuals absent from Thailand during the specified period. The final sample comprised 824 participants, exceeding both the target of 10 observations per indicator variable [65] and the minimum 400-response threshold for population representation [66]. All participants provided voluntary informed consent without compensation, with each individual permitted a single response. Reliability analysis demonstrated strong internal consistency across the three latent variables: digital channels (α = 0.848), public health services (α = 0.958), and resilient cities (α = 0.952), all surpassing the 0.70 reliability benchmark [53].

### Statistical analysis

This study employed a three-phase analytical approach using IBM SPSS 23 and AMOS 23. First, exploratory factor analysis (EFA) was conducted to identify latent constructs through principal component analysis with varimax rotation [67, 68]. Factor extraction retained components with eigenvalues > 1, and items with factor loadings more than 0.5 were considered significant [69]. Second, confirmatory factor analysis (CFA) validated the measurement model of three latent variables, demonstrating: (1) reliability (Cronbach’s α > 0.7; composite reliability (CR) > 0.6) [70], (2) convergent validity (Average Variance Extracted (AVE) ≥ 0.5) [71, 72], and (3) discriminant validity (the absence of redundant items in the measurement model [73]; square roots of AVEs exceeding inter-construct correlations [67, 72]; and all correlations < 0.80) [73, 74]. Finally, structural equation modeling (SEM) evaluated causal relationships using multiple fit indices: (1) the relative chi-square index (CMIN/DF or *χ2*/*df*) < 3.0 [75]; (2) comparative fit index (CFI) > 0.9 [76]; (3) goodness of fit index (GFI) ≥ 0.9 [77]; (4) normed fit index (NFI) ≥ 0.90) [73]; (5) Tucker-Lewis index (TLI) > 0.9 [76]; (6) root mean square error of approximation (RMSEA) < 0.08 [78]; and (7) root mean square residual (RMR) < 0.05 [79]. This comprehensive approach ensured robust validation of the hypothesized relationships between three primary latent variables: digital channels, public health services, and resilient cities.

### Hypotheses development

**Fig. 2:**
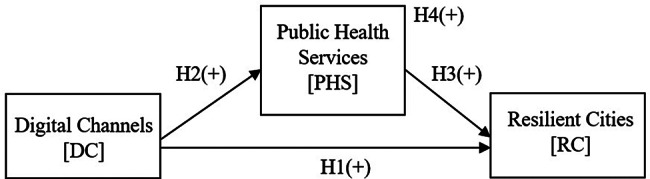
Proposed conceptual model

The proposed conceptual model proposes four hypotheses (H1, H2, H3, and H4) examining relationships between digital channels, public health services, and resilient cities (Fig. 2).

#### H1. Digital channels have a positive effect on resilient cities

Digital channels positively contribute to city resilience by enhancing crisis communication between governments and citizens during PHEs. These platforms facilitate real-time dissemination of critical information about available health services, disease prevention measures, and emergency protocols [33, 37]. By improving public awareness of both health resources and potential risks, digital channels empower populations to navigate service access more effectively, adopt protective behaviors, and mitigate systemic disruptions. This bidirectional information flow strengthens community capacity to adapt to functional failures, ultimately accelerating crisis recovery and the restoration of city normalcy. The combined effect of these mechanisms supports the positive relationship between digital infrastructure and city resilience. This hypothesis is represented as H1+.

#### H2. Digital channels have a positive effect on public health services

Digital channels significantly improve public health service delivery through enhanced data collection from both institutional and community sources, streamlined analysis of health information, and efficient dissemination of service-related data to stakeholders [31, 32, 80]. These digital channels create a bidirectional communication ecosystem where citizens gain improved access to government health resources while simultaneously enabling health authorities to crowdsource community-generated data. This reciprocal exchange empowers individuals to make informed healthcare decisions and participate in public health initiatives, while providing governments with real-time insights for evidence-based service optimization. The resulting synergy between digital infrastructure and participatory engagement elevates the overall quality, responsiveness, and effectiveness of public health services, supporting the positive relationship between these variables. This hypothesis is represented as H2+.

#### H3. Public health services have a positive effect on resilient cities

Effective public health services serve as a critical determinant of city resilience during PHEs by enabling rapid crisis response to minimize systemic disruptions, delivering targeted interventions such as immunization campaigns and outbreak containment protocols to preserve population health, and implementing adaptive service delivery models customized to specific crisis requirements [10, 11, 81]. The velocity and quality of public health interventions exhibit direct proportionality with city recovery timelines, where optimized service delivery enhances a city’s capacity to maintain critical operations during crises, prevent health system collapse through preventive measures, and facilitate post-crisis normalization. This relationship emerges from the compounding benefits of individual health protection, community immunity reinforcement, and institutional preparedness - collectively transforming public health infrastructure into a resilience multiplier during PHE scenarios. This hypothesis is represented as H3+.

#### H4. Digital channels have a positive indirect effect on resilient cities through public health services

Digital channels indirectly strengthen city resilience by optimizing public health service delivery during crises [36, 41, 44]. This mediation occurs through enabling real-time monitoring of population health indicators through data collection and analysis, facilitating evidence-based policy formulation by governments through comprehensive data dissemination, and fostering coordinated citizen engagement in health initiatives. When effectively integrated into public health systems, digital infrastructure transforms service delivery by enhancing responsiveness to emerging threats and enabling targeted interventions. The resulting improvement in health service efficacy measured through effective outbreak containment, more efficient resource allocation, and higher community compliance, subsequently enhances a city’s capacity to absorb shocks and accelerate post-crisis recovery. This cascading effect demonstrates how digital channels serve as critical enablers of resilience when embedded within public health ecosystem. This hypothesis is represented as H4+.

**Fig. 3:**
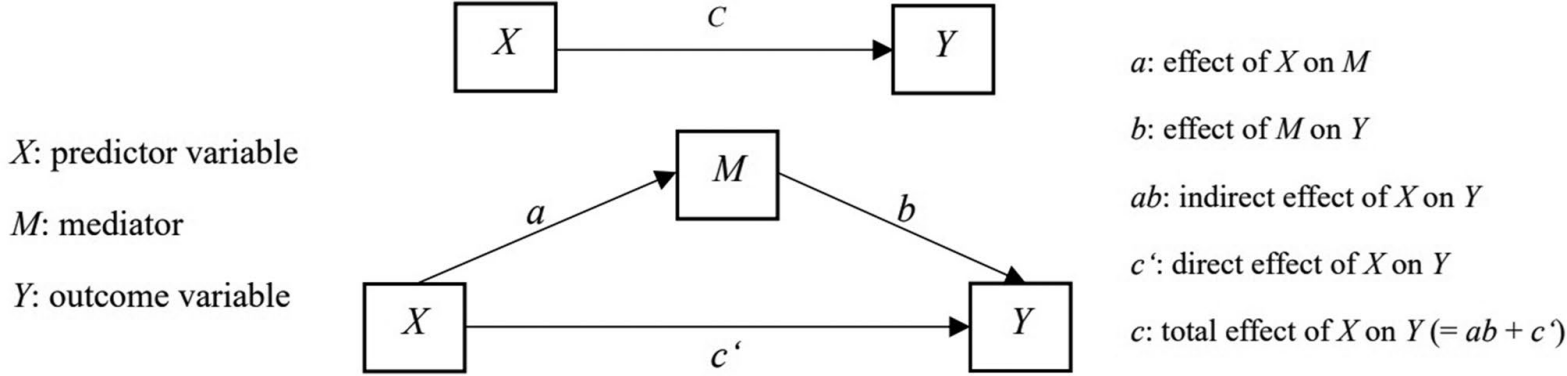
Simple mediation model (based on Demming et al., 2017)

The study evaluated the mediating role of public health services (PHS) between digital channels (DC) and resilient cities (RC) through statistical mediation analysis (Fig. 3) [82]. This approach estimates the indirect effect (ab) of DC on RC through the mediator (PHS), where a statistically significant ab path indicates mediation [82]. Mediation effects are further classified as full mediation or partial mediation: (1) if the direct effect is not significant is full mediation; (2) if the direct effect is significant is partial mediation (If indirect effect and direct effect have opposing signs, it is competitive partial mediation, but if it is not opposing signs, it is complementary partial mediation) [83].

## Results

### Sample Characteristics

The analysis included 824 valid responses, with 98.18% from Thai nationals and 1.82% from foreign residents. Geographically, 25.12% of participants resided in central Thailand. The sample showed nearly equal gender distribution (50.61% male, 49.39% female), with the largest age cohort being 23–42 years (37.50%). Most participants were married (57.52%) and held bachelor’s degrees (46.24%). Professionally, company employees constituted the largest group (29.00%), while the predominant income bracket was 15,001–30,000 Baht (29.98%). Notably, 44.30% of participants reported at least one infection. Complete demographic details are presented in Table 2.

**Table 2:** The demographic characteristics of the participants in Thailand (*n* = 824)

Characteristics	Category	*n*	%
Nationality	Thais	809	98.18
	Foreigners	15	1.82
Gender	Male	417	50.61
	Female	407	49.39
Age	14–22	131	15.90
	23–42	309	37.50
	43–58	300	36.41
	59–77	84	10.19
Marital Status	Single	340	41.26
	Married	474	57.52
	Divorced	10	1.21
Education	Primary School	1	0.12
	Junior High School	9	1.09
	High School	87	10.56
	Diploma	72	8.74
	Bachelor’s Degree	381	46.24
	Master’s Degree	206	25.00
	Doctoral Degree	68	8.25
Occupation	Student	155	18.81
	Self-owned business/Freelance	187	22.69
	Company Employee	239	29.00
	Civil Servant	232	28.16
	Housewife/Housemen	10	1.21
	Other	1	0.12
Monthly Income (Baht)	Less than 5,000	112	13.59
	5,000–15,000	138	16.75
	15,001–30,000	247	29.98
	30,001–50,000	200	24.27
	50,001-85000	88	10.68
	from 85,001	39	4.73
Region	North	165	20.02
	Central	207	25.12
	Northeastern	147	17.84
	East	91	11.04
	West	87	10.56
	South	127	15.41
Infected	More than 3 times	21	2.55
	3 times	45	5.46
	2 times	245	29.73
	1 time	365	44.30
	Never	148	17.96

Note: “infected” describes a state of an individual is impacted by an organism capable of inducing illness, such as viruses

### Characteristics of study variables

#### Exploratory factor analysis

Exploratory Factor Analysis was employed to identify and categorize latent factors, with the Kaiser-Meyer-Olkin (KMO) measure quantifying sampling adequacy for the factor analysis [71, 84] The KMO index ranges from 0 to 1, with minimum acceptable values of 0.6 indicating adequate sampling [73], values between 0.7 and 0.8 representing good suitability [85], and values exceeding 0.8 demonstrating excellent factorability [85]. The KMO test yielded values of 0.824 (digital channels), 0.959 (public health services), and 0.904 (resilient cities), all exceeding the 0.60 threshold and indicating excellent sampling adequacy (Table A). These results suggest strong intercorrelation among variables, with 82%, 96%, and 90% of data gathered, respectively [67]. Bartlett’s Test of Sphericity further validated factorability, showing significant results (*p* < 0.001) with degree of freedom (*df*) of 6, 496, and 120, respectively, and chi-square of 3745.368, 18361.897, and 6004.415, respectively, confirming non-identity matrices [69, 71] Together, these diagnostics demonstrate the data’s appropriateness for factor analysis, meeting all statistical prerequisites for robust dimension reduction.

**Fig. 4:**
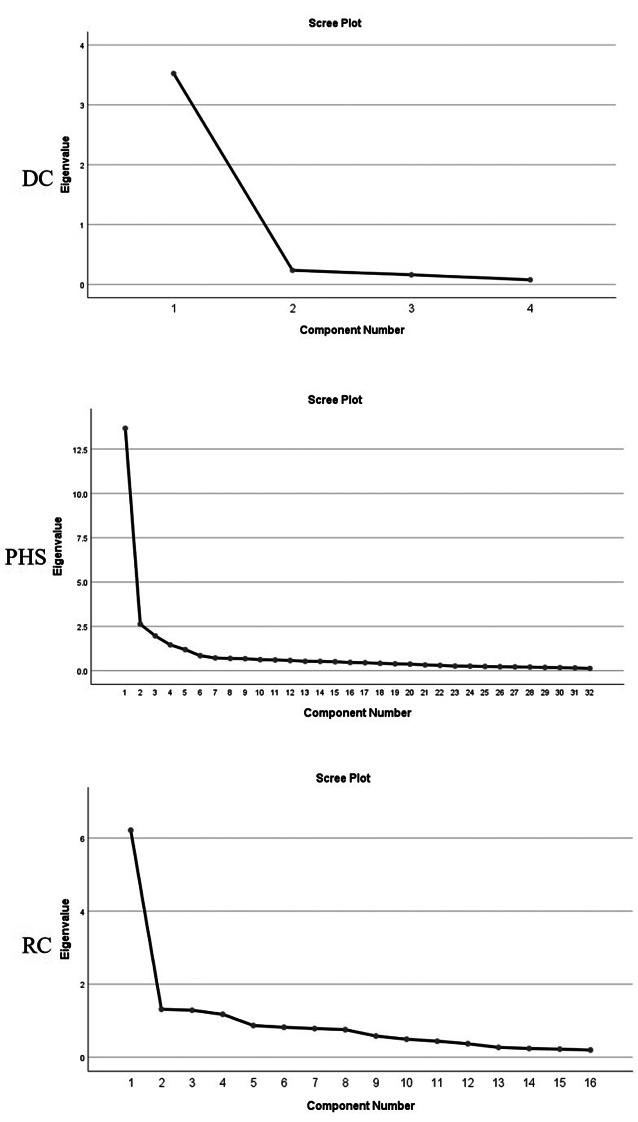
Scree plot of loading of the factors of digital channels (DC), public health services (PHS), and resilient cities (RC)

The study employed varimax rotation to establish factor groupings, retaining only variables demonstrating strong, unambiguous relationships with single factors (factor loadings > 0.50) [86]. This threshold ensures discriminant validity by eliminating cross-loaded or weakly associated items (loadings < 0.50), thereby optimizing the model’s fit [86]. Following rotation, the analysis yielded both expected factor alignments and emergent groupings, necessitating the reclassification and renaming of certain items to accurately reflect their empirical associations. For Digital Channels (DC), a single-component solution emerged with an eigenvalue greater than 1, accounting for 88.121% of the total variance (Table 3). All four DC indicators demonstrated strong factor loadings above 0.50 (Table B), with the scree plot confirming the unidimensional structure (Fig. 4). Public Health Services (PHS) analysis revealed five meaningful components explaining 65.334% of cumulative variance: Service (21.912%), Facility (14.544%), Price (11.799%), Product (10.716%), and Process (6.362%), with all retained indicators exceeding the minimum loading threshold (Table C). Similarly, Resilient Cities (RC) analysis extracted four components representing 62.404% of total variance: Economic Resilience (21.932%), Disaster Resilience (19.730%), Living Resilience (11.935%), and Institutional Resilience (8.808%), with all indicators meeting the 0.50 loading criterion (Table D). The scree plots for DC, PHS and RC analyses confirmed the respective component structures, validating the factor solutions for all three constructs.

**Table 3:** Total variance explained of the factors of DC, PHS, and RC

Total variance explained										
Variables	Component	Initial eigenvalues			Extraction sums of squared loadings			Rotation sums of squared loading		
		Total	% of variance	Cumulative %	Total	% of variance	Cumulative %	Total	% of variance	Cumulative %
DC	1	3.525	88.121	88.121	3.525	88.121	88.121	3.525	88.121	88.121
PHS	1	13.669	42.715	42.715	13.669	42.715	42.715	7.012	21.912	21.912
	2	2.627	8.211	50.926	2.627	8.211	50.926	4.654	14.544	36.456
	3	1.963	6.134	57.060	1.963	6.134	57.060	3.776	11.799	48.256
	4	1.459	4.558	61.618	1.459	4.558	61.618	3.429	10.716	58.972
	5	1.189	3.716	65.334	1.189	3.716	65.334	2.036	6.362	65.334
RC	1	6.214	38.837	38.837	6.214	38.837	38.837	3.509	21.932	21.932
	2	1.312	8.200	47.037	1.312	8.200	47.037	3.157	19.730	41.661
	3	1.285	8.029	55.066	1.285	8.029	55.066	1.910	11.935	53.596
	4	1.174	7.338	62.404	1.174	7.338	62.404	1.409	8.808	62.404

Note. DC, digital channels; PHS, public health services; RC, resilient cities

#### Confirmatory factor analysis

**Fig. 5:**
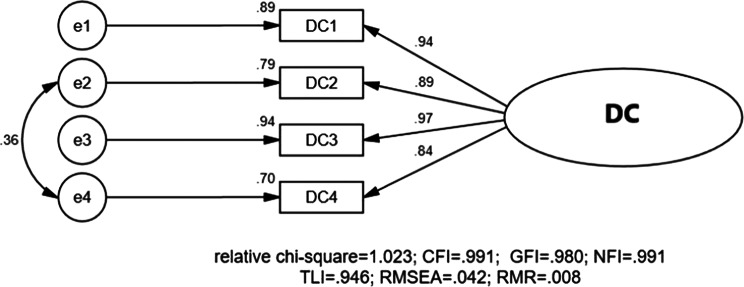
CFA of digital channel (DC) factors after model modification

**Fig. 6:**
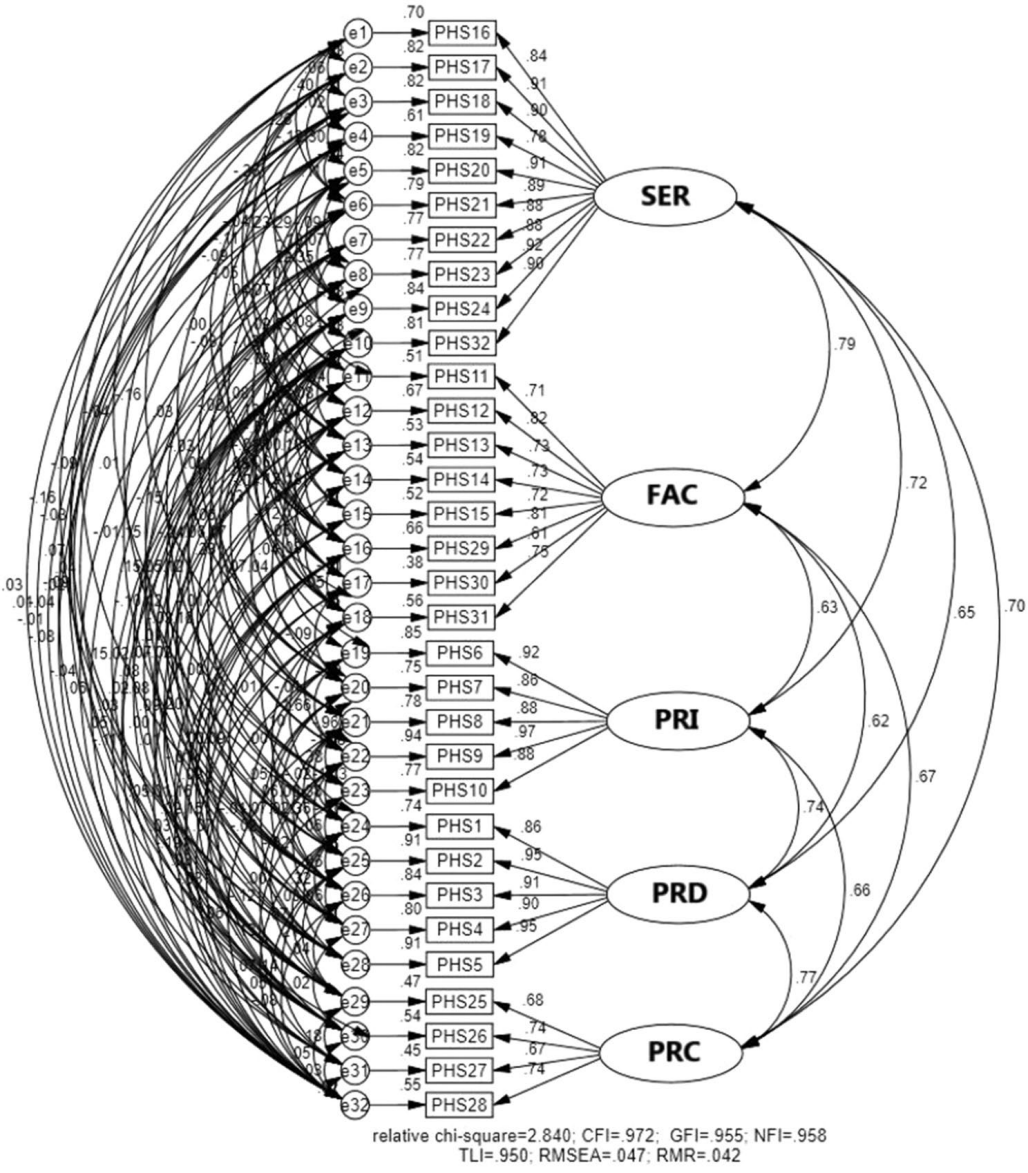
CFA of public health service (PHS) factors after model modification

**Fig. 7:**
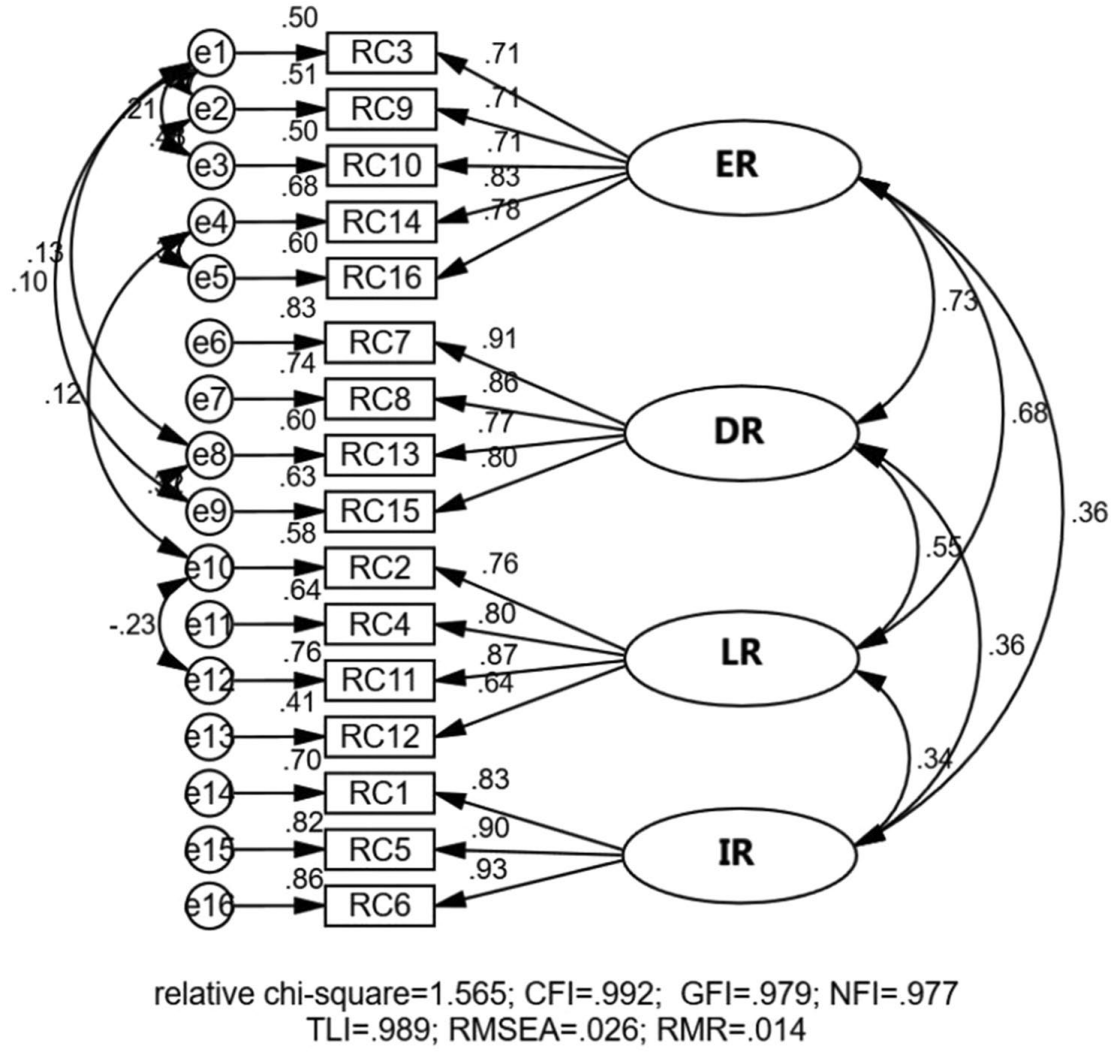
CFA of resilient city (RC) factors after model modification

The measurement model exhibited robust psychometric characteristics, meeting all established thresholds for validity and reliability (Table 4). Reliability was confirmed with α > 0.7 and CR > 0.6 values exceeding established thresholds for all latent variables [70]. Convergent validity was supported by AVE values ≥ 0.5 for each construct [71, 72]. Discriminant validity was established through three complementary tests: (1) the absence of redundant items in the measurement model (Figs. 5, 6 and 7) [73], (2) square roots of AVEs exceeding inter-construct correlations (Table 5) [67, 72], and (3) all factor correlations remaining below 0.80 [73, 74] These results collectively confirm that the measurement model meets rigorous standards for construct validity and reliability [73, 74].

**Table 4:** Measurement reliabilities

Variables	AVE	CR	Cronbach’s alpha	Factor loading				
				Coefficient	S.E.	t	Standard coefficient	*R* ^2^
DC1	0.829	0.951	0.955	1.000	-	-	0.942	0.887
DC2				0.856	0.019	44.581	0.890	0.793
DC3				1.027	0.017	59.104	0.969	0.939
DC4				0.806	0.028	37.223	0.835	0.697
CMIN/DF = 1.023, CFI = 0.991, GFI = 0.980, NFI = 0.991, TLI = 0.946, RMSEA = 0.042, RMR = 0.008								
PHS16	0.775	0.972	0.955	0.720	0.021	34.447	0.780	0.705
PHS17				0.964	0.023	41.752	0.904	0.825
PHS18				0.970	0.021	47.073	0.908	0.817
PHS19				0.668	0.023	29.567	0.839	0.608
PHS20				0.957	0.020	47.294	0.905	0.819
PHS21				0.939	0.024	38.987	0.886	0.785
PHS22				0.982	0.025	38.870	0.880	0.774
PHS23				0.927	0.024	38.124	0.876	0.767
PHS24				0.988	0.023	42.287	0.916	0.839
PHS32				1.000	-	-	0.900	0.811
PHS11	0.546	0.905	0.858	1.000	-	-	0.714	0.510
PHS12				1.200	0.054	22.391	0.818	0.670
PHS13				0.973	0.048	20.267	0.731	0.534
PHS14				1.043	0.051	20.406	0.735	0.540
PHS15				1.029	0.048	21.216	0.722	0.522
PHS29				1.186	0.053	22.217	0.812	0.659
PHS30				0.814	0.046	17.717	0.612	0.375
PHS31				0.970	0.049	19.858	0.750	0.563
PHS6	0.817	0.957	0.901	1.093	0.030	36.853	0.922	0.851
PHS7				0.891	0.027	32.477	0.864	0.747
PHS8				1.062	0.034	31.020	0.882	0.779
PHS9				1.288	0.044	28.980	0.970	0.942
PHS10				1.000	-	-	0.877	0.769
PHS1	0.839	0.963	0.931	0.834	0.022	38.529	0.859	0.738
PHS2				1.049	0.017	61.659	0.954	0.910
PHS3				0.916	0.018	51.521	0.914	0.836
PHS4				0.865	0.018	46.756	0.896	0.804
PHS5				1.000	-	-	0.953	0.908
PHS25	0.503	0.802	0.728	0.995	0.049	20.200	0.685	0.469
PHS26				0.892	0.043	20.726	0.737	0.543
PHS27				1.019	0.044	22.950	0.673	0.453
PHS28				1.000	-	-	0.739	0.546
CMIN/DF = 2.840, CFI = 0.972, GFI = 0.955, NFI = 0.958, TLI = 0.950, RMSEA = 0.047, RMR = 0.042								
RC3	0.561	0.864	0.889	0.848	0.047	18.179	0.710	0.504
RC9				0.982	0.054	18.263	0.714	0.510
RC10				0.948	0.052	18.139	0.709	0.503
RC14				1.083	0.037	29.568	0.826	0.682
RC16				1.000	-	-	0.777	0.604
RC7	0.699	0.902	0.909	1.211	0.042	29.037	0.909	0.825
RC8				1.142	0.041	27.538	0.861	0.742
RC13				0.971	0.033	29.389	0.772	0.596
RC15				1.000	-	-	0.795	0.632
RC2	0.595	0.853	0.800	1.000	-	-	0.759	0.577
RC4				0.848	0.051	8.720	0.800	0.639
RC11				0.804	0.072	9.827	0.871	0.759
RC12				0.898	0.059	13.455	0.638	0.407
RC1	0.792	0.919	0.796	1.762	0.386	4.563	0.834	0.819
RC5				2.072	0.459	4.516	0.904	0.863
RC6				1.000	-	-	0.929	0.802
CMIN/DF = 1.565, CFI = 0.992, GFI = 0.979, NFI = 0.977, TLI = 0.989, RMSEA = 0.026, RMR = 0.014								

Note. DC, digital channels; PHS, public health services; RC, resilient cities

**Table 5:** Correlations between constructs

	DC	SER	FAC	PRI	PRD	PRC	ER	DR	LR	IR
DC	0.910									
SER	0.444**	0.880								
FAC	0.401**	0.574**	0.739							
PRI	0.367**	0.657**	0.392**	0.904						
PRD	0.439**	0.707**	0.660**	0.572**	0.916					
PRC	0.210**	0.210**	0.173**	0.276**	0.209**	0.709				
ER	0.357**	0.428**	0.450**	0.262**	0.395**	0.125**	0.749			
DR	0.310**	0.482**	0.469**	0.459**	0.465**	0.206**	0.634**	0.836		
LR	0.352**	0.445**	0.548**	0.320**	0.520**	0.094**	0.497**	0.456**	0.771	
IR	0.127**	0.125**	0.177**	0.107**	0.147**	0.161**	0.205**	0.210**	0.178**	0.890

Note.**Correlation is significant at the 0.01 level (2-tailed)

DC, digital channels; SER, service, FAC, facility; PRI, price; PRD, product; PRC, process; ER, economic resilience; DR, disaster resilience; LR, living resilience; IR, institutional resilience

#### Structural equation modeling

Path coefficients were computed to examine directional relationships between latent variables in the structural model. DC served as the exogenous variable (independent of other model variables), while PHS and RC functioned as endogenous variables (influenced by other model variables) [87]. These path coefficients, representing either positive (+) or negative (-) relationships, were used to statistically test the study’s hypotheses regarding these variable interactions. Path analysis revealed significant positive relationships across all hypotheses (Tables 6, 7 and 8), with standardized coefficients (*β*) interpreted as: *β* < 0.30 (weak), 0.30 ≤ *β* ≤ 0.60 (moderate), and *β* > 0.60 (strong) [88]. Specifically, DC demonstrated a weak but significant direct effect on RC (*β* = 0.19, *p* < 0.001; supporting H1) and a strong direct effect on PHS (*β* = 0.70, *p* < 0.001; supporting H2). PHS showed a strong direct effect on RC (*β* = 0.69, *p* < 0.001; supporting H3), while DC exhibited a moderate indirect effect on RC through PHS (*β* = 0.48, *p* < 0.001; supporting H4).

**Table 6:** Path’s coefficient and their significance

Hypotheses	Paths	Standard path coefficient	*p*-value	Status
H1(+)	DC → RC	0.192	***	Confirmed
H2(+)	DC → PHS	0.698	***	Confirmed
H3(+)	PHS → RC	0.686	***	Confirmed

Note. DC, digital channels; PHS, public health services; RC, resilient cities

***p < 0.001

**Table 7:** Results of the causal influence of the structural equation modeling

Dependent variables	R^2^	Influence	Independent variables	
			DC	PHS
PHS	0.488	Direct Effect	0.698***	-
		Indirect Effect	-	-
		Total Effect	0.698***	-
RC	0.691	Direct Effect	0.192***	0.686***
		Indirect Effect	0.479***	-
		Total Effect	0.671***	0.686***

Note. DC, digital channels; PHS, public health services; RC, resilient cities

The sign - means there is no parameter of the hypotheses

***p < 0.001

**Table 8:** Result of mediating variable measurement

Model	Standard coefficient
Direct Effect	
DC → RC (*c*)	0.671***
Indirect Effect	
DC → PHS (*a*)	0.698***
PHS → RC (*b*)	0.686***
DC → RC (*c‘*)	0.192***
Indirect Effect (*a* x *b*)	0.479***

Note. DC, digital channels; PHS, public health services; RC, resilient cities

***p < 0.001

**Table 9:** Validity measurement and factor loading of latent variables

Primary latent variables	Indicators/dimensions	Coefficient	S.E.	t	Standard coefficient	*R* ^2^
DC	DC1	1.000	-	-	0.956***	0.914
	DC2	0.834	0.023	35.688	0.858***	0.737
	DC3	1.016	0.022	46.598	0.949***	0.900
	DC4	0.820	0.024	34.073	0.841***	0.707
PHS	SER	0.800	0.049	13.762	0.835***	0.698
	FAC	0.793	0.042	18.709	0.840***	0.706
	PRI	0.770	0.037	21.053	0.743***	0.552
	PRD	1.248	0.041	30.709	0.898***	0.807
	PRC	1.000	-	-	0.759***	0.576
RC	ER	0.800	0.068	11.213	0.839***	0.704
	DR	1.051	0.044	23.799	0.862***	0.744
	LR	0.819	0.050	16.497	0.826***	0.683
	IR	1.000	-	-	0.799***	0.639

Note. DC, digital channels; PHS, public health services; RC, resilient cities

CMIN/DF = 2.189, CFI = 0.954, GFI = 0.900, NFI = 0.919, TLI = 0.946, RMSEA = 0.038, RMR = 0.047

***p < 0.001

**Fig. 8:**
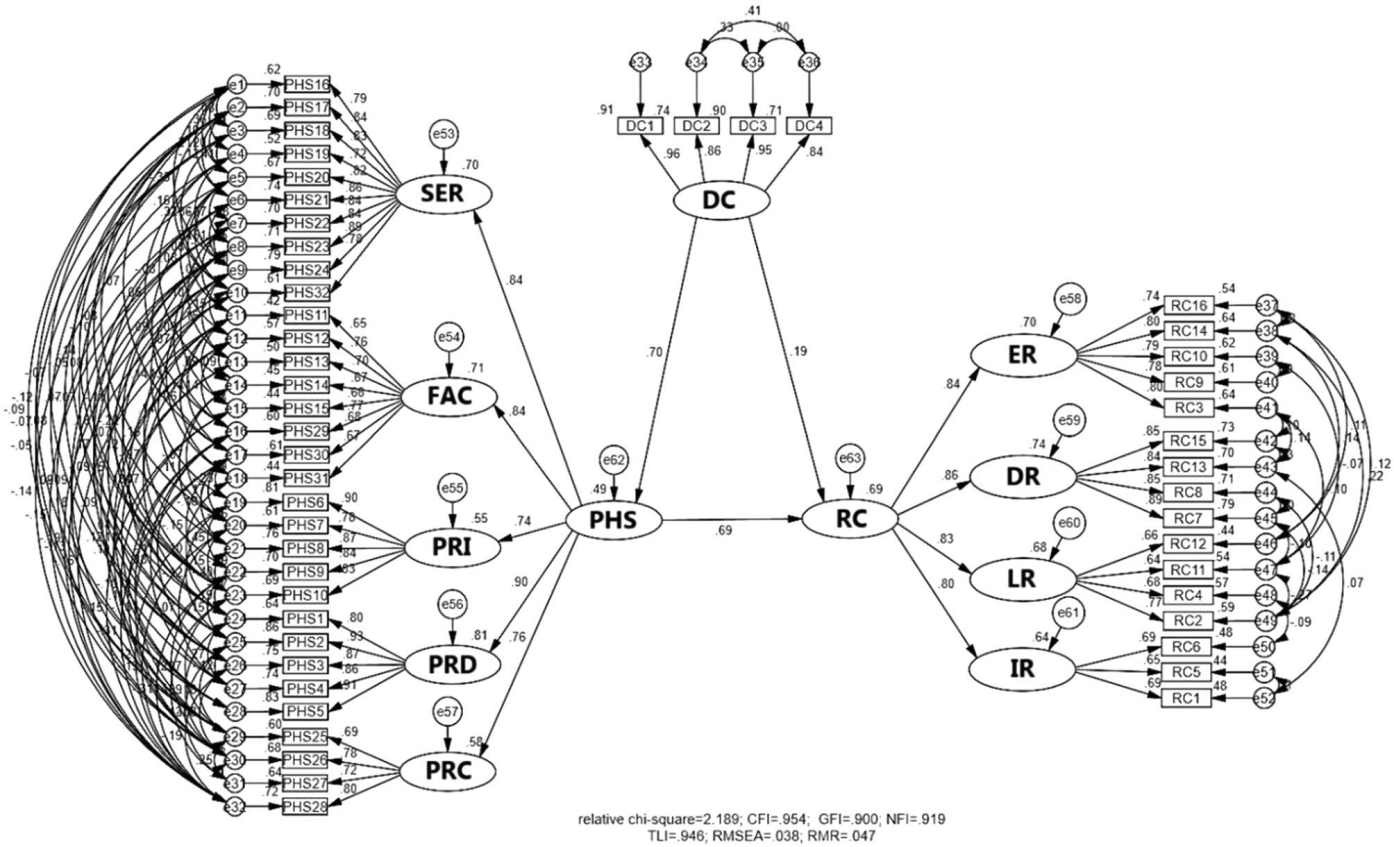
The graphics results of the structural model

As illustrated in Table 9; Fig. 8, the structural equation model examining the impact of digital channels on public health services to enhance city resilience during the PHE response in Thailand (2020–2023) demonstrated excellent fit with the empirical data. The model passed all the required criteria: *X*^*2*^/*df* = 2.189, CFI = 0.954, GFI = 0.900, NFI = 0.919, TLI = 0.946, RMSEA = 0.038, and RMR = 0.047. These results confirm that the final modified model validly represents the hypothesized relationships among three primary latent variables.

## Discussion

This study empirically validated a conceptual model elucidating how digital channels strengthen city resilience through enhanced public health service delivery, employing a robust analytical approach (EFA, CFA, and SEM).

### Indicators of digital channels

EFA identified four key components explaining 88.121% of the total variance in digital channel performance. These components—websites, mobile applications, social media platforms, and television channels—serve as measurable indicators of digital channel effectiveness. The high explained variance confirms that these four factors are the indicators of digital channels.

### Indicators and dimensions of public health services

The application of EFA to public health service evaluation yielded five robust dimensions—service, facility, price, product, and process—that systematically influence overall performance outcomes during the PHE.

The ‘Service’ dimension, explaining 21.912% of total variance, comprises ten key indicators measuring digital-enabled public health communication: (1) publicizing knowledge about vaccination, treatment, and self-protection; (2) using social media platforms to provide information about virus infection; (3) using mobile applications to increase communication channels; (4) promoting or campaigning for vaccination or infection test; (5) updating of public health service delivery quality (performance); (6) accessing information, advice, or suggestions from volunteers; (7) accessing information, advice, or suggestions from government doctors or nurses; (8) accessing information, advice or suggestions from non-government or government pharmacists; (9) accessing information, advice or suggestions from government officials from other agencies; and (10) accessing details, pictorial, or video information on mobile apps or websites provided by the government. The ten indicators listed above can effectively measure the service dimension.^15,27,46^ These indicators demonstrate three core functional capacities: first, the systematic circulation of critical health data including prevention protocols and service outcomes; second, enabling multichannel engagement between the public and health professionals; and third, ensuring equitable geographic access through digital platforms that transcend physical limitations. The dimension’s emphasis on comprehensive information management—from data collection to public dissemination—establishes a framework for evidence-based health decision-making during public health emergencies, particularly through the strategic use of official health applications and online media channels [46].

The ‘Facility’ dimension, accounting for 14.544% of total variance, comprises eight indicators assessing the spatial and operational adequacy of health infrastructure: (1) the infection test sites are available across the country; (2) vaccination sites are available across the country; (3) pharmacies and medicine stores are available across the country; (4) treatment facilities or hospitals are available across the country; (5) quarantine facilities are available across the country; (6) signs or logos of infection test facilities, vaccination facilities, treatment facilities, or quarantine facilities; (7) internal and external environment of the healthcare facilities; and (8) medical equipment in the healthcare facilities. These eight indicators effectively measure the facility dimension [17, 27]. These indicators collectively evaluate four critical aspects of service accessibility: geographic coverage of essential health nodes, intuitive wayfinding through visual identifiers, resource adequacy (equipment/supplies), and therapeutic environment design. The dimension emphasizes that effective public health service delivery requires not only widespread facility distribution but also optimized user experience through thoughtful spatial planning and resource allocation.

The ‘Price’ dimension explains 11.799% of total variance and comprises five key indicators measuring financial transparency in public health services: (1) information about service prices; (2) information about medical product prices; (3) information about available options to receive treatment or other services; (4) information about available choices of treatment prices or other services prices such as quarantine; and (5) information about payment methods. These indicators collectively assess how pricing structures influence service effectiveness through four mechanisms: cost transparency, service affordability, benefit inclusivity, and payment flexibility. The dimension highlights that equitable health service delivery depends not only on clinical quality but also on financial accessibility and clear communication of economic considerations [15–17].

The ‘Product’ dimension accounts for 10.716% of total variance and comprises five essential indicators measuring critical medical resources: (1) information about medical supplies (masks and alcohol for hand sanitizers); (2) information about vaccines; (3) information about medicines; (4) ATK; and (5) information about medical equipment for RT-PCR testing. These five indicators effectively measure the product dimension [12, 13]. These indicators demonstrate that comprehensive product information dissemination directly enhances public health service effectiveness by enabling informed self-protection behaviors. The dimension underscores how transparent communication about medical resources—their availability, proper usage, and accessibility—serves as a fundamental component of successful public health interventions during emergencies [89].

The ‘Process’ dimension, explaining 6.362% of total variance, comprises four key operational indicators: (1) registration (book an appointment) for vaccines, treatments, and services; (2) steps for receiving vaccines, treatments, and services; (3) details about recommendations of before and after of vaccination, treatments, and services; and (4) accessing personal health information. These indicators collectively evaluate the efficiency and clarity of service delivery mechanisms, providing the public with transparent procedural knowledge to facilitate compliance. The dimension highlights how well-structured operational workflows—from digital appointment scheduling to clear instructional guidance—significantly enhance the usability and effectiveness of public health services during emergencies [15, 20, 21].

### Indicators and dimensions of resilient cities

In analyzing the dimensions that impact the resilience of cities. The results from the EFA method show four dimensions that are influencing city resilience.

EFA identified ‘Economic Resilience’ as the primary dimension influencing city resilience, accounting for 21.932% of total variance. This dimension measures a city’s capacity to adapt to, recover from, and mitigate disruptions to core economic functions during emergencies [62, 90]. Five key indicators operationalize this construct: (1) ability to access the internet; (2) ability to buy or sell products or services online; (3) ability to do financial transaction online; (4) ability to communicate and do social activities through online (such as seminar, meeting, or other online activities); and (5) ability to access learning resources online. Thailand’s pandemic response demonstrated these capacities through sustained economic activity despite social distancing measures. The ubiquitous internet infrastructure enabled continuous economic interactions—benefiting both producers and consumers—by supporting remote transactions, communication, and learning. These indicators collectively validate that digital connectivity serves as the foundational pillar of economic resilience during public health crises.

The ‘Disaster Resilience’ dimension accounts for 19.730% of total variance, measuring the collective capacity of governments, organizations, and communities to prepare for, adapt to, and recover from emergencies [91–93]. This dimension comprises four critical indicators: (1) the government can continue responding to the PHE; (2) the coordination of the central government and other government agencies in responding to the PHE; (3) following the disease prevention and control measures recommended by the government; and (4) ability of people in sharing knowledge or information about self-protection to others. These metrics demonstrate three synergistic resilience mechanisms: institutional continuity (government response maintenance), systemic collaboration (cross-agency coordination), and social capital (public adherence and information dissemination). Thailand’s pandemic experience revealed that effective disaster resilience emerges when policy frameworks (government planning), operational networks (interagency cooperation), and civic engagement (preventive behavior adoption) function cohesively. The dimension particularly highlights how public participation in preventive measures and peer education complements institutional response efforts, creating a multi-layered defense system against health crises.

The ‘Living Resilience’ dimension accounts for 11.935% of total variance, reflecting a population’s capacity to maintain essential daily activities during crises [94–96]. This dimension comprises four measurable indicators: (1) ability to travel or utilize public transportation; (2) ability to employ water, electricity, gas, and fuel; (3) ability to receive salary or income; and (4) having food, drinking water, clothing, medicine, and housing. These indicators demonstrate Thailand’s success in preserving fundamental living standards during the pandemic, evidenced by sustained public mobility, uninterrupted utility services, stable income streams, and consistent availability of essential goods. The dimension underscores the critical infrastructure and economic systems that enable populations to withstand disruptions while maintaining baseline quality of life.

The ‘Institutional Resilience’ dimension explains 8.808% of total variance, measuring organizational capacity to adapt to disruptions and maintain functionality during PHEs [97]. This dimension evaluates three core capabilities: (1) ability to work or study online; (2) the organizations can continue their work; and (3) the organizations can cooperate with other organizations. These three indicators demonstrate Thailand’s institutional adaptability during the pandemic, where organizations successfully leveraged digital infrastructure to sustain productivity while establishing cooperative networks to mitigate systemic shocks. The dimension highlights how resilient institutions serve as critical stabilizers during crises by ensuring service continuity, workforce engagement, and cross-sector coordination despite operational constraints [61].

### Digital channels, public health services, and resilient cities

With the structural model, the regression path estimate was established based on the correlation coefficients among three primary latent variables: digital channels (DC), public health services (PHS), and resilient cities (RC). Sub-objective 1 to examine the impact of digital channels on public health services is answered by the result of hypothesis 2 (H2). Sub-objective 2 to examine the impact of public health services on resilient cities is answered by the result of hypothesis 3 (H3), and sub-objective 3 to examine the impact of digital channels on resilient cities is answered by the result of hypotheses 1 and 4 (H1 and H4).

The analysis reveals a strong positive direct effect of digital channels (DC) on public health services (PHS), supported by a significant path coefficient (*β* = 0.70, *p* < 0.001), supporting H2. This finding aligns with prior studies done by Sun and Wang (2022), Yu (2022), and Chu et al. (2021), demonstrating digital channels’ critical role in optimizing public health service delivery through streamlined data collection and analysis, enhanced government-public communication, and improved accessibility of health service information [31, 32, 80] Specifically, digital channels facilitate bidirectional exchange—governments disseminate service information while citizens contribute situational data—creating a collaborative framework for health crisis management. The public accesses PHS resources across five key dimensions via digital platforms: product (0.898), facility (0.840), service (0.835), process (0.759), and price (0.743). These results collectively underscore how digital infrastructure empowers evidence-based decision-making at both institutional and individual levels during PHEs.

The analysis confirms a strong positive relationship between public health services (PHS) and resilient cities (RC), with a significant path coefficient (*β* = 0.69, *p* < 0.001), supporting H3. This finding aligns with established studies of Stoto et al. (2017), Olu (2017), and Rose et al. (2017), emphasizing the critical role of efficient public health systems in post-crisis recovery [10, 11, 81]. This study demonstrates that effective public health service delivery plays a critical role in restoring disrupted city functions, including daily life operations, institutional workflows, disaster response systems, and economic stability. The analysis reveals a direct correlation between efficient public health administration and accelerated city recovery. Thailand’s SARS-CoV-2 response (2020–2023) illustrates how optimized service delivery—particularly in pricing structures, service accessibility, facility management, process operation, and product distribution—not only addressed immediate crisis needs but also established adaptable frameworks for future pandemic management. These public health interventions simultaneously supported population health through immunity enhancement and wellness preservation while strengthening systemic city resilience. The cumulative effect of these measures enabled cities to achieve faster, more sustainable recovery from PHEs.

The study confirms a modest but significant positive relationship between digital channels (DC) and resilient cities (RC), with a significant path coefficient (*β* = 0.19, *p* < 0.001), supporting H1. This finding aligns with prior studies of Qiu et al. (2022), Yao et al. (2023), and George et al. (2023), demonstrating how digital platforms facilitate critical crisis communication through three key mechanisms: enhancing public awareness of health services, improving risk perception of diseases, and enabling efficient dissemination of official health information [33, 37]. While digital channels serve as vital infrastructure for government-public communication—particularly regarding public health services—their effectiveness in building city resilience depends on complementary factors beyond information exchange alone. The analysis reveals that digital tools achieve greatest impact when paired with active civic engagement and institutional responsiveness, creating a synergistic relationship where information sharing informs practical adaptation strategies. This study demonstrates that digital dissemination of government public health information positively impacted city resilience during the PHE, albeit at a low level. While these findings underscore the importance of knowledge sharing, they also reveal its limitations. Effective PHE responses require active participation from both governmental institutions and the public.

This study confirms that digital channels (DC) indirectly enhance resilient cities (RC) through public health services (PHS), with a significant mediated effect (*β* = 0.479, *p* < 0.001), as PHS serves as a complementary partial mediator between DC and RC, supporting H4. These findings corroborate earlier studies by Mohideen (2021), Raj et al. (2021), and Zou (2024), who supported that digital channels serve as vital instruments for advancing health solutions, significantly improving public health service delivery, while the strategic provision of these services constitutes a critical step in restoring normalcy [41]. Digital channels strengthen city resilience through three synergistic mechanisms: first, by enabling data-driven public health service delivery optimization through real-time monitoring of health needs and dynamic resource allocation; second, by fostering governance-public synergy through bidirectional information flows where authorities disseminate health protocols and citizens contribute situational data; and third, by empowering behavioral adaptation through accessing to service information across all dimensions of public health services [36]. This mediation pathway explains how digital channels help cities recover systemic functions during crises—not through direct effects but by making public health service delivery more responsive through predictive analytics, participatory governance, and precision communication.

Through EFA and CFA, items were classified by factor loading strength: strong (≥ 0.6), moderate (0.4–0.6), and weak (< 0.4) [98]. Items with higher factor loadings show higher participant perception, demonstrating successful translation of policy into practice. Conversely, lower factor loadings suggest participants perceived these operational elements as lower, reflecting inconsistent or limited implementation in practice.

The modification model used the following testing tools to test the overall model’s fitness. The *χ*2/*df* value is 2.189 met the recommended value of < 3.0 as suggested by Hu and Bentler 1999 [75]. CFI was 0.954 which is within the recommended threshold of > 0.9 as recommended by Joreskog and Sorbom 1996 [76]. The GFI value is 0.900 which is within the acceptable threshold of ≥ 0.9 as mentioned by Al-Ghamdi et al. 2023 [77]. NFI value is 0.919 which falls within the acceptable range of ≥ 0.9 as indicated by Othman et al. 2014 [73]. TLI value is 0.946 which is within the recommended threshold of > 0.9 as endorsed by Joreskog and Sorbom 1996 [74]. RMSEA value is 0.038 which meets the recommended value of < 0.08 as proposed by Steiger 1990 [78]. Last, the RMR value is 0.047 which met the recommended < 0.05 as remarked by Byrne 2013 [79]. Collectively, these comprehensive fit indices confirm that the modified model not only satisfies all necessary criteria but demonstrates exceptional correspondence with the empirical data, establishing its validity for substantive interpretation.

The findings provide policymakers with evidence-based insights to prioritize digital infrastructure development for crisis communication, optimize public health service delivery through technology integration, and design targeted interventions that leverage digital channels to accelerate city recovery during future PHEs. Implementing these strategies helps cities sustain essential services during crises and restore normal operations faster.

### Thailand national strategic plan for emerging infectious disease

In accordance with Sect. 9 of the Emergency Decree on Public Administration in Emergency Situations B.E. 2548 (2005), Thailand implemented prevention and control measures during the PHE. The government disseminated critical public health information through multiple digital channels, including official websites, social media platforms, mobile applications, and television channels. Two particularly significant mobile applications were deployed: the “Mor Prom” application streamlined vaccination management by enabling appointment scheduling, aggregating nationwide vaccination data, and displaying test results (both RT-PCR from hospitals and ATK from clinics/pharmacies) [99]. It also introduced a “Digital Health Pass” feature - a QR code-based health certification system issued by the Ministry of Public Health for domestic travel verification. Complementing this, the “Thai Chana” platform (developed by Krungthai Bank and launched May 2020) provided venue-based contact tracing through QR code check-in/check-out functionality at commercial establishments, significantly enhancing the nation’s capacity to identify transmission clusters and monitor potentially exposed individuals [100]. Together, these digital initiatives formed a comprehensive technological infrastructure supporting Thailand’s multifaceted pandemic response strategy.

### Limitations and future research

This study acknowledges several methodological and conceptual limitations. First, the research design may inadvertently exclude vulnerable populations, including elderly individuals and those with limited digital literacy or rural residency, who face barriers in accessing digital communication channels. This sampling constraint extends to the online questionnaire format, which inherently excludes non-digital users. To address this limitation, public health agencies should complement digital dissemination with non-digital outreach strategies during emergencies. Second, while the study examines digital channels’ roles in data collection, analysis, and dissemination, it does not differentiate the unique functional capacities of specific platforms (e.g., television’s unilateral dissemination versus mobile apps’ interactive capabilities). Third, the reliance on convenience and snowball sampling may limit the generalizability of findings, potentially underrepresenting demographic groups whose perspectives could differ substantially from the participant pool. Fourth, although back-translation procedures with bilingual experts addressed linguistic equivalence, the Thai-language pilot testing may introduce cultural biases for international participants. Fifth, the analysis omits potentially influential variables such as socioeconomic status and digital literacy. Sixth, the dataset lacks granularity to analyze urban-rural disparities or digital literacy differentials in channel utilization.

Future research should investigate: (a) policy effectiveness across diverse emergency contexts (e.g., floods, tsunamis); (b) vaccine service systems encompassing development, distribution, and adverse event management; (c) community-level health interventions through village volunteer networks; and (d) expanded resilience metrics capturing additional city dimensions. These directions would strengthen both theoretical models and practical applications of digital-enabled public health systems.

### Policy implication

To enhance public health service effectiveness, policymakers should implement two complementary strategies: systematically easing control measures across product distribution, facility accessibility, service delivery, process optimization, and pricing structures, while strategically leveraging digital channels for integrated data collection, analysis, and dissemination. These measures collectively strengthen city resilience by maintaining essential operations during crises. Policy development should consider multidimensional impacts across city functions, including disaster response, economic stability, daily living conditions, and institutional capacity. Crucially, the intersection of streamlined health services with robust digital infrastructure creates an adaptive governance model that minimizes societal disruption while enabling efficient restoration of normalcy. This approach particularly benefits public health emergency management (PHEM) by aligning operational flexibility with data-driven decision-making, ensuring both immediate crisis response and sustainable long-term recovery.

## Conclusions

This study establishes a comprehensive model demonstrating how digital channels enhance city resilience through improved public health services during Thailand’s SARS-CoV-2 response (2020–2023). Analyzing 824 validated responses from Thai and foreign residents through SPSS 23 and AMOS 23.

The research confirms four key hypotheses regarding these relationships. The findings identify five operational dimensions of public health services (product: five indicators [12, 13], facility: eight indicators [22], service: ten indicators [19], process: four indicators [20], and price: five indicators) [18], and four dimensions of resilient cities (disaster resilience: four indicators^91–93^, economic resilience: five indicators [62, 90], living resilience: four indicators [95, 96], and institutional resilience: three indicators) [97], all facilitated through four indicators of digital channels (websites, mobile applications, social media platforms, and television channels) [24, 27–29]. The validated modification model reveals significant causal pathways where digital channels utilization strengthens public health service delivery in data management (data collection, analysis, and dissemination), which in turn bolsters resilient cities. Thailand’s pandemic response demonstrated this framework’s practical application, where digital access to public health service information across five dimensions enabled maintenance of critical city functions despite crisis conditions. The public’s engagement with government-provided digital health resources proved instrumental in preserving disaster response capabilities, economic stability, quality of life, and institutional operations throughout the emergency period.

This research makes three substantive contributions: (1) developing a comprehensive assessment framework comprising 32 validated public health service indicators and 16 resilient city indicators specifically designed for PHE contexts, (2) theoretically explicating the stabilization role of digitally-enhanced PHS during crises, and (3) providing actionable recommendations for policymakers (utilizing the PHS framework), technology partners (co-developing secure platforms), and city planners (applying RC indicators). The study ultimately demonstrates that strategic integration of digital infrastructure with public health systems creates mutually reinforcing resilience capacities, offering both immediate crisis adaptation and sustainable recovery pathways.

## Supplementary Information

Below is the link to the electronic supplementary material.

Supplementary Material 1

Supplementary Material 2

## Data Availability

All data generated or analyzed during this study are included in this published article [and its supplementary information files].
