# Supplementary material for: The impact of digital channels on public health services to enhance city resilience during the public health emergency response in Thailand (2020–2023)
Source: BMC Health Serv Res. 2025 Sep 30;25:1249. doi: 10.1186/s12913-025-13480-4 (PMC12482637; doi:10.1186/s12913-025-13480-4)
Supplement: Supplementary file 1 — Supplementary Material 1 [file 12913_2025_13480_MOESM1_ESM.pdf]

Survey Questionnaire

This questionnaire is for academic purposes only. It is for the thesis topic “The Impact of Digital Governance on City Resilience Development in Thailand—A Case Study of Public Health Emergency Response." The questionnaire primarily examines how digital governance contributes to the development of city resilience in the event of a public health emergency (PHE). This study is a component of a PhD thesis from the Department of Urban Management and Development, School of Public Affairs, Zhejiang University, People's Republic of China. Your submission of any information will be treated as private and used exclusively for research and academic purposes. Please feel free to share your information.

I sincerely appreciate your help.

Watcharaporn Chutarong,

A PhD candidate, School of Public Affairs,

Zhejiang University.

Explanation

This questionnaire is divided into four sections as follows: Section 1 - Basic Information; Section 2 - Digital Channels; Section 3 - Public Health Services; and Section 4 - Resilient Cities.

Note: The public health emergency response in this questionnaire is indicative of the SARS-CoV-2 response in Thailand (2020-2023).

Section 1: Basic Information

Please tick/click the number that corresponds with your answer.

1. Nationality

   ☐ 1) Thai ☐ 2) Other................................

2. Gender

   ☐ 1) Male ☐ 2) Female

3. Age

   ☐ 1) 14-22 ☐ 2) 23-42 ☐ 3) 43-58 ☐ 4) 59-77

4. Which part of Thailand did you live in during the public health emergency response?

   ☐ 1) North ☐ 2) Central ☐ 3) North-East

   ☐ 4) East ☐ 5) West ☐ 6) South

5. How long have you lived in Thailand during the public health emergency response?

   ☐ 1) 6-12 months ☐ 2) more than 1 year but less than 2 years ☐ 3) more than 2 years

6. Marital Status

   ☐ 1) Single ☐ 2) Married ☐ 3) Divorced

7. Education Status

   ☐ 1) Primary School ☐ 2) Junior High School ☐ 3) High School

☐ 4) Diploma ☐ 5) Bachelor's degree ☐ 6) Master's degree

☐ 7) Doctoral degree

8. Occupation

☐ 1) Student ☐ 2) Self-owned business/Freelance ☐ 3) Company employee

☐ 4) Civil servants ☐ 5) Housewife/Housemen ☐ 6) Others……………………………

9. Monthly income (THB) during the public health emergency response

☐ 1) Less than 5,000 ☐ 2) 5,000-15,000 ☐ 3) 15,001-30,000

☐ 4) 30,001-50,000 ☐ 5) 50,001-85,000 ☐ 6) from 85001

10. How many times that you had infected?

☐ 1) more than 3 times ☐ 2) 3 times ☐ 3) 2 times ☐ 4) 1 time ☐ 5) 0 (never)

Section 2: Digital Channels

Please tick/click the number that corresponds with your answer.

5 means "Strongly Agree" and 1 means "Strongly Disagree". A higher score means a higher level of attitude.

Question	5	4	3	2	1
	Strongly Agree	Agree	Neutral	Disagree	Strongly Disagree
During the public health emergency, you accessed data, information, or activities about the public health emergency response from the government with these channels.					
1. Websites					
2. Social Media Platforms					
3. Mobile Applications					
4. Television Channels					

Section 3: Public Health Services

Please tick/click the number that corresponds with your answer.

5 means "Strongly Agree" and 1 means "Strongly Disagree". A higher score means a higher level of attitude.

Question	5	4	3	2	1
	Strongly Agree	Agree	Neutral	Disagree	Strongly Disagree
**During the public health emergency, you accessed the following information or activities from the government through the use of websites/social media platforms/mobile applications/television channels.**					
1) Product					
1. How to use medical supplies to protect yourself from infections, including masks and alcohol usage					
2. Information about vaccines					
3. Information about medicines					
4. How to test for infection using the Antigen Test Kit (ATK)					

Question	5<br>Strongly Agree	4<br>Agree	3<br>Neutral	2<br>Disagree	1<br>Strongly Disagree
5. How to test for infection using real time polymerase chain reaction (RT-PCR)					
**2) Price**					
1. Service prices such as infection tests, and quarantine					
2. Medical product prices, such as vaccines, and medicines					
3. The available options to receive treatments or other services at a reduced cost					
4. The available choices of treatment prices or other service prices, such as quarantine					
5. Payment Methods					
**3) Place**					
1. The infection test sites were available across the country					
2. Vaccination sites were available across the country					
3. Pharmacies and medicine stores were available across the country					
4. Treatment facilities/hospitals were available across the country					
5. Quarantine facilities were available across the country					
**4) Promotion**					
1. Publicized knowledge about vaccination/treatments/self-protection					
2. Social media platforms provided information about virus infection					
3. Mobile applications increased communication channels					
4. Promotion or campaign for vaccination/infection test					
5. Update of public health service delivery quality (performance)					
**5) People**					
1. Information/advice/suggestions from volunteers					
2. Information/advice/suggestions from government doctors or nurses					
3. Information/advice/suggestions from non-government/government pharmacists					
4. Information/advice/suggestions from government officials from other agencies					
**6) Process**					
1. Registration (book an appointment) for vaccines/treatments/services					
2. Steps for receiving vaccines/treatments/other services					
3. Before and after information about vaccination/treatments/services					
4. Online personal health information (name, age, gender, congenital diseases, etc.)					
**7) Physical Evidence**					
1. Signs/logos of infection test facilities/vaccination facilities/treatment facilities/quarantine facilities.					
2. Internal and external environment of healthcare facility					
3. Medical equipment in healthcare facility					
4. Details, pictorial, or video information on mobile apps/websites provided by the government					

Section 4: Resilient Cities

Please tick/click the number that corresponds with your answer.

5 means “Strongly Agree” and 1 means “Strongly Disagree”. A higher score means a higher level of attitude.

During the use of easing measures [the easing control measures include lockdown cancellation, school resumption, reopening of restaurants, business facilities, and borders (land, air, and sea)].

Question	5 Strongly Agree	4 Agree	3 Neutral	2 Disagree	1 Strongly Disagree
**During the use of easing control measures, you did the following activities as usual (normally)**					
**1) Infrastructural Resilience**					
1. Online work or study					
2. Public transportation usage					
3. Internet accessing					
4. Access to water, electricity, gas, and fuel					
**2) Institutional Resilience**					
1. The organizations function normally					
2. The coordination between organizations					
3. Continuous government’s response to the emergency					
4. Cooperation of the central government and other government agencies in response to the emergency					
**3) Economic Resilience**					
1. Buy/sell products or services online					
2. Do financial transaction online					
3. Normal wage/salary/income (financial status)					
4. Basic amenities (food, drinking water, clothing, medicine, and housing)					
**4) Social Resilience**					
1. Follow to preventive measures recommended by the government					
2. Communicate and do social activities through online (seminars, meetings, and other activities)					
3. Share knowledge or information about self-protection to others					
4. Access learning resources online					

Suggestions: ....

....

....
