# Supplementary material for: The impact of digital channels on public health services to enhance city resilience during the public health emergency response in Thailand (2020–2023)
Source: BMC Health Serv Res. 2025 Sep 30;25:1249. doi: 10.1186/s12913-025-13480-4 (PMC12482637; doi:10.1186/s12913-025-13480-4)
Supplement: Supplementary file 2 — Supplementary Material 2 [file 12913_2025_13480_MOESM2_ESM.pdf]

Appendix

Table A KMO and Bartlett's test result.

KMO and Bartlett's Test		
Digital Channels (DC)		
Kaiser-Meyer-Olkin Measure of Sampling Adequacy.		.824
Bartlett's Test Sphericity	Approx. Chi-Square	3745.368
	df	6
	Sig.	.000
Public Health Services (PHS)		
Kaiser-Meyer-Olkin Measure of Sampling Adequacy.		.959
Bartlett's Test Sphericity	Approx. Chi-Square	18361.897
	df	496
	Sig.	.000
Resilient Cities (RC)		
Kaiser-Meyer-Olkin Measure of Sampling Adequacy.		.904
Bartlett's Test Sphericity	Approx. Chi-Square	6004.415
	df	120
	Sig.	.000

Table B Rotated factor matrix of factors in DC.

Item	Factor Loading
DC1	.946
DC2	.940
DC3	.954
DC4	.915

Table C Rotated factor matrix of factors in PHS.

Item	Factor loading				
	1	2	3	4	5
PHS1				.731	
PHS2				.712	
PHS3				.693	
PHS4				.706	
PHS5				.703	
PHS6			.818		
PHS7			.791		
PHS8			.769		
PHS9			.618		
PHS10			.772		
PHS11		.680			
PHS12		.674			
PHS13		.647			
PHS14		.677			

PHS15		.670			
PHS16	.802				
PHS17	.777				
PHS18	.799				
PHS19	.759				
PHS20	.763				
PHS21	.735				
PHS22	.704				
PHS23	.730				
PHS24	.660				
PHS25					.713
PHS26					.638
PHS27					.667
PHS28					.671
PHS29		.647			
PHS30		.630			
PHS31		.631			
PHS32	.754				

Table D Rotated factor matrix of factors in RC.

Item	Factor loading			
	1	2	3	4
RC1				.650
RC2			.614	
RC3	.697			
RC4			.621	
RC5				.648
RC6				.665
RC7		.839		
RC8		.811		
RC9	.797			
RC10	.834			
RC11			.600	
RC12			.696	
RC13		.790		
RC14	.729			
RC15		.801		
RC16	.729			
